# Clinical characteristics and mortality associated with COVID-19 at high altitude: a cohort of 5161 patients in Bogotá, Colombia

**DOI:** 10.1186/s12245-022-00426-4

**Published:** 2022-05-21

**Authors:** David Rene Rodriguez Lima, Ángela María Pinzón Rondón, Cristhian Rubio Ramos, Darío Isaías Pinilla Rojas, Maria José Niño Orrego, Mateo Andrés Díaz Quiroz, Nicolás Molano-González, Jorge Enrique Ceballos Quintero, Alex Francisco Arroyo Santos, Ángela María Ruiz Sternberg

**Affiliations:** 1 Critical and Intensive Care Medicine, Hospital Universitario Mayor-Méderi, Bogotá, Colombia; 2 Grupo de Investigación Clínica, Escuela de Medicina y Ciencias de la Salud, Universidad del Rosario, Bogotá, Colombia; 3 Planning Department, Hospital Universitario Mayor-Méderi, Bogotá, Colombia

**Keywords:** Altitude, COVID-19, SARS-CoV-2, Mortality, Clinical outcomes

## Abstract

**Background:**

There are few data on the clinical outcomes of patients with coronavirus disease 2019 (COVID-19) in cities over 1000 m above sea level (masl).

**Objectives:**

To describe the clinical characteristics and mortality of patients with COVID-19 treated at a high complexity hospital in Bogotá, Colombia, at 2640 masl.

**Methods:**

This was an observational study of a cohort including 5161 patients with confirmed COVID-19 infection from 19 March 2020 to 30 April 2021. Demographic data, laboratory values, comorbidities, oxygenation indices, and clinical outcomes were collected. Data were compared between survivors and nonsurvivors. An independent predictive model was performed for mortality and invasive mechanical ventilation (IMV) using classification and regression trees (CART).

**Results:**

The median cohort age was 66 years (interquartile range (IQR) 53–77), with 1305 patients dying (25%) and 3856 surviving (75%). The intensive care unit (ICU) received 1223 patients (24%). Of 898 patients who received IMV, 613 (68%) of them perished. The ratio of partial pressure arterial oxygen (PaO_2_) to fraction inspired oxygen (FiO_2_), or the P/F ratio, upon ICU admission was 105 (IQR 77–146) and 137 (IQR 91–199) in the deceased and survivors, respectively. The CART model showed that the need for IMV, age greater than 79 years, ratio of oxygen saturation (SaO_2_) to FiO_2_, or the S/F ratio, less than 259, and lactate dehydrogenase (LDH) greater than 617 U/L at admission were associated with a greater probability of death.

**Conclusion:**

Among more than 5000 patients with COVID-19 treated in our hospital, mortality at hospital discharge was 25%. Older age, low S/F ratio, and high LDH at admission were predictors of mortality.

## Background

Epidemiological data on risk factors and clinical outcomes related to severe acute respiratory syndrome coronavirus 2 (SARS-CoV-2) infection and the associated clinical entity (i.e. COVID-19) have emerged from all over the world; however, there are limited data on the clinical outcomes of patients with COVID-19 living in cities over 1000 masl.

According to the Wilderness Medicine Society, regions are classified according to altitude as high altitude when they are located at more than 2500 masl [1]. More than 140 million people in the world live above 2500 masl, with this being more frequent in the Latin American Andean region [2]. With higher altitudes come a lower barometric pressure (BP), and the FiO_2_ remains constant (20.93%) at any altitude. The pressure of inspired oxygen (PiO_2_) is positively correlated with BP. At sea level (*B*P = 760 mmHg), the PiO_2_ is 159.1 mmHg [3], while in Bogotá (2640 masl; *BP* = 560 mmHg), it is 117.2 mmHg. Lower PiO_2_ leads to lower alveolar oxygen pressure and PaO_2_, causing tissue hypoxia [4]. The average PaO_2_ in Bogotá is 67 mmHg [5]. This phenomenon is known as hypobaric hypoxaemia. Despite this, prolonged exposure to hypobaric hypoxaemia allows adaptation of the respiratory, cardiovascular, haematological, muscular, and microcirculatory systems to optimize the cellular supply and use of oxygen [6].

High altitude has effects on the burdens of chronic diseases. Convincing data from population studies show that living above 2000 masl reduces mortality from coronary heart disease and stroke [7], in addition to the risk of developing obesity and diabetes [8, 9]. It is speculated that, at altitude, cold induces thermogenesis and decreases appetite, and there is an unintentional increase in physical activity and a better tolerance to glucose related to hypoxaemia [10]. Each of these phenomena represents an explanatory mechanism of the inverse relationship between altitude and cardiovascular diseases. These findings are compatible with those reported by Seclén et al. [11] who, in an ecological study, found the incidence and mortality of patients with COVID-19 in 25 Peruvian regions with altitudes between 1 and 3744 masl, finding an inverse relationship between the prevalence of diabetes, obesity, and hypertension with altitude.

It is known that high altitude has varying effects on other respiratory conditions, such as influenza pneumonia and tuberculosis. Influenza pneumonia at high altitude is associated with higher mortality, stating that hypobaric hypoxaemia directly increases the risk of serious disease and requires early treatment with oxygen [12], whereas pulmonary tuberculosis at high altitude shows lower mortality compared to rates at sea level, which is associated with an increase in cellular antimycobacterial immunity [13].

Arias-Reyes et al. [14] raised the hypothesis that living at high altitude can protect against the effects of SARS-CoV-2 infection based on the observation of the low severity and prevalence of COVID-19 in regions located at more than 3000 masl in Tibet, Bolivia, and Ecuador. Cano-Pérez et al. [15] in Colombia analysed data from 70 municipalities with altitudes between 1 and 3180 masl, finding an inverse relationship in the case fatality rate of COVID-19 and altitude level. Data from other ecological studies support these observations [16–18]. This can be explained via physiological adaptations of the angiotensin-converting enzyme (ACE) system [19]. SARS-CoV-2 enters human cells through the binding of its spike protein to ACE-2; then, it is internalized, and its replication begins. ACE-2 is widely present in human organs, with high expression in the respiratory tract, vascular endothelium, myocardium, renal tubules, gastrointestinal tract, testicles, and central nervous system, which explains the multisystemic involvement of severe COVID-19 disease [20]. Arias-Reyes et al. [14] also proposed that SARS-CoV-2 may be less virulent at high altitude owing to hypoxia-induced downregulation of ACE-2 expression, thus limiting viral replication and organ compromise.

However, information is contradictory. Zhang et al. and Dang et al. found a decrease in ACE-2 in cellular and animal models with hypoxaemia [21, 22]; however, other studies in different models have reported that hypoxia upregulates ACE-2 [23, 24]. The potential negative or positive clinical impact of ACE-2 upregulation or downregulation by oxygenation levels in COVID-19 has yet to be determined [20]. More recent additional ecological studies also do not support the positive effect of high altitude on clinical results. Valverde-Bruffau et al. [25], after analysing 1122 municipalities in Colombia, did not find a protective effect between the altitude and severity of COVID-19 disease, findings similar to those of Cardenas et al. [26] who, in 2881 municipalities of Peru and Colombia with altitudes between 2 and 4705 masl, found no difference in mortality.

The current evidence does not allow us to ascertain whether hypobaric hypoxaemia can prevent, treat, or aggravate COVID-19 infection [27].

This study was carried out in the city of Bogotá, capital of Colombia, located 2640 masl, with 8,848,588 inhabitants. By June 2021, more than 1,382,000 COVID-19 cases and 25,000 deaths had been reported https://saludata.saludcapital.gov.co/osb/index.php/datos-de-salud/enfermedades-trasmisibles/covid19/.

The aim of this study was to describe the clinical characteristics, laboratory findings, and mortality of patients with COVID-19 treated at the Hospital Universitario Mayor Méderi located in Bogotá, Colombia.

## Methods

### Study design and participants

This was an observational, single-centre, cohort study that included all patients with COVID-19 admitted to the Hospital Universitario Mayor Méderi in Bogotá, Colombia, conducted between 19 March 2020 and 30 April 2021. This is a high complexity university hospital located in the centre of the city of Bogotá, at 2640 masl. It has 99 intensive care beds, which during the pandemic were expanded to 131 beds, with 94 exclusively for the care of patients with COVID-19. It also has 790 general hospital beds, of which 295 are used for the care of patients with COVID-19. SARS-CoV-2 infection was confirmed by real-time reverse transcription-polymerase chain reaction (RT-PCR) in throat swab samples. We excluded individuals with incomplete medical records and patients under 16 years of age.

### Data collection

We reviewed clinical electronic medical records and laboratory findings for all patients with laboratory-confirmed SARS-CoV-2 infection. On admission, we collected the following data: age, sex, past medical history, days of hospital stay, days of ICU stay, body mass index (BMI), management site (outpatients, inpatients, ICU, or emergency patients), laboratory values on admission (leucocyte blood count, neutrophil-lymphocyte ratio (NLR), haematocrit, haemoglobin, LDH, D-dimer, procalcitonin, ferritin), and oxygenation indices (S/F ratio at admission and at 48 h, P/F ratio at admission, 48 h, and at ICU admission). As is routine, electronic medical data were archived onto a local server, from which we retrieved these data.

### Outcomes

The clinical outcomes assessed were discharge or death, need for IMV, and site management. For this study, 4 management sites were defined as follows:

1. Outpatients: those who consulted and were discharged in the first 24 h and were not readmitted in the following 3 weeks.
2. Inpatients: those who were admitted to the hospitalization service, with a stay > 24 h and did not transfer to the ICU at any time during their hospital stay.
3. Intensive care unit (ICU): those who were admitted to the ICU at any time during their hospital stay.
4. Emergency department (ED): those who received care only in the ED.

### Statistical analysis

Qualitative variables were reported as frequencies and percentages, while quantitative variables were reported as medians and interquartile ranges. Clinical, sociodemographic, and laboratory characteristics were compared in relation to mortality, mechanical ventilation requirements, and management site (emergency room, ICU, hospitalization, or outpatient management) using nonparametric Mann-Whitney *U*-tests for all continuous variables (assuming heterogeneous variance) and *X*^2^ tests for categorical variables. We fitted a classification and regression tree (CART) using all the collected variables for comparison purposes. Selection of the covariates included for CART modelling was based on their biological and clinical relevance, on what was previously reported in the literature, and their statistical significance in the bivariate analysis. The CART algorithm quantified each variable’s weight and built risk profiles. This methodology contrasts with classical regression models in that the CART algorithm can discover effective modifiers and complex interactions between variables, whereas in regression models, the researcher must provide those interactions based on a prior hypothesis. Statistical analysis was performed using R software version 4.1.0 (Free Software Foundation’s GNU Public Licence), and the CART were modelled using the package RPART (recursive partition and regression trees).

## Results

The cohort included 5161 adult patients treated at the Hospital Universitario Mayor Méderi. Patients who were still hospitalized by 30 April 2021 and 1 patient in whom the review of the electronic medical record was not possible were excluded from the final analysis. In total, 321 patients were excluded.

Of the 5161 patients, 2340 (45%) of them were women. A total of 1305 patients died (25%), and 3856 survived to hospital discharge (75%). The median age was 66 years (IQR 53–77). All patients were identified as Latino.

The most frequent comorbidity was arterial hypertension (23%), followed by hypothyroidism (7%), chronic obstructive pulmonary disease (COPD) (5.6%), and diabetes mellitus (5%).

Table 1 shows the baseline characteristics of the patients and the bivariate analysis in relation to mortality.

**Table 1:** Baseline characteristics and bivariate analysis by mortality

Variable	Overall patients (***n*** = 5161)		Alive (***n*** = 3856)		Death (***n*** = 1305)		***p***-value
	Patients	Missing (***n***)	Patients	Missing (***n***)	Patients	Missing (***n***)	
**Demographic**							
Age median (IQR)	66 (53–77)	0	62 (48–73)	0	76 (67–84)	0	< 0.01*
Days of hospital stay (IQR)	8 (4–13)	0	7 (3–12)	3356	11 (5–17)	655	< 0.01*
Days of ICU (IQR)	9 (4–15)	3951	8 (4–14.5)	3313	9 (5–15)	638	0.016*
Body mass index median (IQR)	26.34 (23.46–29.38)	3	26.44 (23.71–29.41)	2	25.84 (23.18–29.35)	1	< 0.01*
Sex							
Female	2340 (45.34%)	0	1850 (79.06%)	0	490 (20.94%)	0	< 0.01*
Male	2821 (54.65%)	0	2006 (71.11%)	0	815 (28.89%)	0	< 0.01*
**Management site**							
Inpatient	2970 (57.54%)	0	2459 (82.79%)	0	511 (17.21%)	0	< 0.01*
ICU	1223 (23.69%)	0	543 (44.40%)	0	680 (55.60%)	0	< 0.01*
Outpatient	854 (16.54%)	0	854 (100.00%)	0	0 (0%)	0	< 0.01*
Emergency	114 (2.20%)	0	0 (0%)	0	114 (100%)	0	< 0.01*
**Comorbidities**							
Hypertension	1171 (22.68%)	0	803 (68.57%)	0	368 (31.43%)	0	< 0.01*
Diabetes types 1 and 2	256 (4.96%)	0	163 (63.67%)	0	93 (36.33%)	0	< 0.01*
COPD	290 (5.61%)	0	158 (54.48%)	0	132 (45.52%)	0	< 0.01*
HIV	9 (0.17%)	0	7 (77.78%)	0	2 (22.22%)	0	-
Hypothyroidism	358 (6.93%)	0	247 (68.99%)	0	111 (31.01%)	0	0.011*
**Laboratory values median (IQR)**							
Leukocyte blood count	7,985 (0.2–262.9)	439	7.75 (5.76–10.51)	415	8.62 (6.22–12.02)	24	< 0.01*
Neutrophil-lymphocyte ratio	6,0303 (3,4258–10,7008)	442	5.391 (3.131–9.519)	415	8,322 (4,634–14,896)	27	< 0.01*
Haematocrit	43.4 (39.2–47)	434	43.7 (39.9–47.1)	410	42.5 (36.9–46.7)	24	< 0.01*
Haemoglobin	14.5 (13–15.8)	433	14.6 (13.3–15.8)	410	14.1 (12.2–15,675)	23	< 0.01*
LDH	337 (255–445)	765	321 (247–410)	661	400 (288–549)	104	< 0.01*
D-Dimer	950 (540–1802.25)	1013	857 (497–1490)	855	1296 (721.5–2641.5)	158	< 0.01*
Procalcitonin	0.31 (0.12–1.01)	3592	0.2 (0.09–0.57)	2918	0.63 (0.24–2,225)	674	< 0.01*
Ferritin	881 (478–1612.5)	4834	789.5 (417.75–1422,25)	3642	1225 (594–2359)	1192	< 0.01*
**Oxygenation values median (IQR)**							
S/F ratio at admission	339.28 (310.71–423.80)	166	346,429 (321,429–428,571)	159	318,452 (184–361,905)	7	< 0.01*
S/F ratio at 48 h	325 (254.28–341.52)	806	328,571 (303,333–342,857)	690	281.25 (167,273–328,571)	116	< 0.01*
P/F ratio at admission	233,3854 (180,9524–274,6429)	711	242,381 (202.5–279,524)	655	191,786 (112.8–254,643)	56	< 0.01*
S/F ratio at ICU admission	168 (105.55–255.55)	*4011	186 (121.25–288,281)	3356	149,615 (95,667–194)	655	< 0.01*
P/F ratio at ICU admission	118.14 (81.55–171.44)	*4049	137.4 (91,089–198,917)	3378	105 (76,706–146)	671	< 0.01*

*ICU* intensive care unit, *COPD* chronic obstructive pulmonary disease, *HIV* human immunodeficiency virus, *LDH* lactate dehydrogenase, *S/F* oxygen saturation to fraction inspired oxygen, *P/F* partial pressure of oxygen to fraction inspired oxygen

^*^P-value refers to statistically significant result

A total of 114 patients (2%) were managed in the ED, with 114 deaths (100%). In all, 39 of these patients (34%) were ventilated and died before being transferred to the ICU, and 75 patients (66%) were admitted for conservative management but remained in the emergency expansion area of the ED until their deaths due to high hospital occupancy. This high mortality can be explained given that the median age in this group was 81 years (IQR, 72–85, 75).

In all, 854 patients (17%) received outpatient management without reporting any mortality in the national registry system the month after discharge; these patients were the youngest in the cohort, with a median age of 47 years (IQR, 31–64.75).

A total to 2970 (58%) participants were inpatients, with 511 deaths (17%). The median age in this group was 68 years (IQR, 56–79). The high number of patients who died while hospitalized without being admitted to the ICU is explained by the elderly characteristics of the group, as the median age was 83 years (IQR, 74.5–88.0). Out of these 511 patients, 8 (0.02%) were ventilated and died before being transferred to the ICU, and the 503 remaining inpatients, together with the family and the guidelines of the institutional ethics committee, decided to limit invasive manoeuvres, such as orotracheal intubation or transfer to the ICU.

A total of 1223 participants (24%) were admitted to the ICU, with 680 deaths (56%). Of the 1223 ICU admissions, 851 of them were ventilated (70%). Out of the ventilated group, 566 of them died (67%) vs. 114 (31%) in the nonventilated group. These patients were admitted to the ICU but did not want orotracheal intubation, and this intervention was not performed. It is clarified that some patients were admitted to the ICU to receive noninvasive mechanical ventilation and haemodynamic support without orotracheal intubation, which was possible only at moments without the pandemic peaking. In Bogotá, for the date of the performance of this analysis, May 2021, there were 2 peaks with high hospital occupancy, the first in July and August 2020 and the second in December 2020 and January 2021, at which time only patients were admitted to the ICU with requirements of IMV.

Forty-seven patients received IMV and did not enter the ICU, along with 39 patients in the emergency room and 8 patients in hospitalization; these patients died before being transferred to the ICU because they were so sick. A total of 898 patients (17%) received IMV in this cohort, of whom 613 died (68%).

Table 2 shows the bivariate analysis in relation to the management site, and Table 3 shows the bivariate analysis in relation to the requirement for IMV.

**Table 2:** Bivariate analysis by management site

Variable	Overall (***n*** = 5161)	Outpatient (***n*** = 854)		Inpatient (***n*** = 2970)		Emergency room (***n*** = 114)		ICU (***n*** = 1223)		***p***-value
	Median (IQ)	Median (IQ)	Missing (***n***)	Median (IQ)	Missing (***n***)	Median (IQ)	Missing (***n***)	Median (IQ)	Missing (***n***)	
**Demographic**										
Age median (IQR)	66 (53–77)	47 (31–64.7)	0	68 (56–79)	0	81 (71–85.7)	0	69 (59–76)	0	< 0.01*
Days of hospital stay (IQR)	8 (4–13)	1 (0–2)	0	7 (5–11)	0	6 (1–14)	0	15 (10–24)	0	< 0.01*
Days of ICU (IQR)	-	-	-	-	-	-	-	9 (4–15)		-
Body mass index median (IQR)	26.34 (23.4–29.38)	25.7 (23.3–28.8)	2	26.15 (23.4–28.8)	0	23.7 (23–29.3)	1	27 (24.2–30.8)	0	< 0.01*
Sex										
Female	2340 (45.34%)	489 (20%)	-	1370 (58%)	-	42 (1.7%)	-	439 (18%)	-	< 0.01*
Male	2821 (54.65%)	365 (12%)	-	1600 (56%)	-	72 (2.5%)	-	784 (27%)	-	< 0.01*
**Comorbidities**										
Hypertension	1171 (22.68%)	82 (7.0%)	-	723 (61%)	-	27 (2.3%)	-	339 (28%)	-	< 0.01*
HIV	9 (0.17%)	1 (11%)	-	7 (77%)	-	0 (0%)	-	1 (11%)	-	-
COPD	290 (5.61%)	21 (7.2%)	-	179 (61%)	-	14 (4.8%)	-	76 (26%)	-	< 0.01*
Diabetes types 1 and 2	256 (4.96%)	25 (0.04%)	-	138 (0.2%)	-	10 (0.01%)	-	83 (0.1%)	-	< 0.01*
**Laboratory values (IQR)**										
Leukocyte blood count	7,985 (0.2–262.9)	6.72 (5.23–9.11)	387	7.74 (5.72–10.42)	33	8.31 (5.99–11.82)	17	9.18 (6.68–12.9)	2	< 0.01*
Neutrophil-lymphocyte ratio	6,0303 (3,4258–10,7008)	3.84 (2.3–6.68)	387	5.54 (3.2–9.71)	35	9.1 (5.7–17.63)	17	8.52 (4.8–14.74)	3	< 0.01*
Haematocrit	43.4 (39.2–47)	44.1 (41–47.3)	386	43.2 (39–46.8)	29	42.1 (36–45.7)	17	43.7 (39.2–47.4)	2	< 0.01*
Haemoglobin	14.5 (13–15.8)	14.7 (13.7–15.9)	386	14.4 (12.9–15.7)	28	14.1 (11.6–15.3)	17	14.6 (13–15.9)	2	< 0.01*
D-Dimer	950 (540–1802.25)	755 (430–1253)	518	914 (530–1703)	347	1506 (962.7–3420.2)	24	1078 (585–2071)	124	< 0.01*
LDH	337 (255–445)	293 (217–361.75)	448	318 (246–406)	218	429 (283.5–580.5)	23	420 (311–554.5)	76	< 0.01*
Ferritin	881 (478–1612.5)	825 (615–1680)	817	786 (400–1492)	2765	1415 (826.5–1690)	106	1162 (705–2027)	1146	< 0.04*
Procalcitonin	0.31 (0.12–1.01)	0.2 (0.08–0.54)	794	0.17 (0.09–0.53)	2288	0.57 (0.21–3.95)	79	0.49 (0.19–1.7)	431	< 0.01*
**Outcome**										
Alive	3856 (74%)	854 (22%)	-	2459 (63%)	-	0 (0%)	-	543 (14%)	-	< 0.01*
Dead	1305 (25%)	0 (0%)	-	511 (39%)	-	114 (8.7%)	-	680 (52%)	-	< 0.01*
**Oxygenation indices (IQR)**										
S/F ratio at admission	339.28 (310.71–423.80)	433.3 (357.4–447.6)	159	339.2 (321.4–414.2)	0	278 (138.5–335.7)	5	321.4 (188–371.4)	2	< 0.01*
S/F ratio at 48 h	325 (254.28–341.52)	328.5 (293–328.5)	617	328.5 (314.2–346.4)	113	268.7 (170–325)	41	240.8 (156.6–325)	35	< 0.01*
P/F ratio at admission	233,3854 (180,9524–274,6429)	256 (216.7–293.2)	488	243.3 (203.8–279.5)	192	175.5 (92–227.7)	20	188.9 (106.8–247.6)	11	< 0.01*
S/F ratio at ICU admission	-	-	-	-	-	-	-	168.5 (105.5–255.5)	73	-
P/F ratio at ICU admission	-	-	-	-	-	-	-	118.14 (81.5–171.4)	111	-

*ICU* intensive care unit, *COPD* chronic obstructive pulmonary disease, *HIV* human immunodeficiency virus, *LDH* lactate dehydrogenase, *S/F* oxygen saturation to fraction inspired oxygen, *P/F* partial pressure of oxygen to fraction inspired oxygen

^*^P-value refers to statistically significant result

**Table 3:** Bivariate analysis for invasive mechanical ventilation

Variable	Overall (***n*** = 5161)	No requirement of invasive mechanical ventilation (***n*** = 4263)	Missing (***n***)	Invasive mechanical ventilation (***n*** = 898)	Missing (***n***)	***p***-value
**Demographic**						
Age median (IQR)	66 (53–77)	65 (51–78)	0	68 (59–75)	0	< 0.01*
Days of ICU (IQR)		4 (2–7)	3891	11 (7–18)	60	< 0.01*
Days of hospital stay (IQR)	8 (4–13)	7 (3–11)	0	16 (10–25)	0	< 0.01*
Body mass index median (IQ)	26.34 (23.4–29.38)	26.1 (23.4–29.2)	3	27.3 (24.3–31.1)	0	< 0.01*
Sex						
Female	2340 (45.34%)	2036 (87%)	-	304 (12%)	-	< 0.01*
Male	2821 (54.65%)	2227 (78%)	-	594 (21%)	-	< 0.01*
**Management site**						
Outpatient	854 (16.54%)	854 (100%)	-	0 (0%)	-	-
Inpatient	2970 (57.54%)	2962 (99%)	-	8 (0.002%)	-	-
Emergency	114 (2.20%)	75 (65%)	-	39 (34%)	-	-
ICU	1223 (23.69%)	372 (30%)	-	851 (70%)	-	-
**Outcome**						
Alive	3856 (75%)	3571 (92%)	-	285 (0.7%)	-	< 0.01*
Dead	1305 (25%)	692 (53%)	-	613 (46%)	-	< 0.01*
**Comorbidities**						
Hypertension	1171 (22.68%)	935 (79%)	-	236 (20%)	-	< 0.01*
HIV	9 (0.17%)	8 (88%)	-	1 (11%)	-	-
COPD	290 (5.61%)	232 (8%)	-	58 (2%)	-	-
Diabetes mellitus	256 (4.96%)	193 (0.37%)	-	63 (0.12%)	-	< 0.01*
**Laboratory values (IQR)**						
Leukocyte blood count	7,985 (0.2–262.9)	7.7 (5.72–10.49)	432	9.27 (6.7–12.95)	7	< 0.01*
Neutrophil-lymphocyte ratio	6,0303 (3,4258–10,7008)	5,436 (3,168–9,674)	434	9,378 (5,399–16,548)	8	< 0.01*
Haematocrit	43.4 (39.2–47)	43.3 (39.2–46.9)	427	43.9 (39.4–47.5)	7	< 0.01*
Haemoglobin	14.5 (13–15.8)	14.4 (13–15.7)	426	14.7 (13.1–16)	7	< 0.01*
LDH	337 (255–445)	317 (243–407)	725	453.5 (341–590)	40	< 0.01*
D-Dimer	950 (540–1802.25)	920 (523.2–1711.5)	937	1093 (600–2028)	76	< 0.01*
Procalcitonin	0.31 (0.12–1.01)	0.2 (0.09–0.58)	3347	0.58 (0.23–1.92)	245	< 0.01*
Ferritin	881 (478–1612.5)	816 (462–1538)	4002	1244 (79.7–1925.7)	832	< 0.01*
**Oxygenation indices (IQR)**						
S/F ratio at admission	339.28 (310.71–423.80)	342,857 (321.4–428.57)	163	300 (161–347)	3	< 0.01*
S/F ratio at 48 h	325 (254.28–341.52)	328.57 (307.1–346.4)	777	202.2(145–317)	29	< 0.01*
P/F ratio at admission	233,3854 (180,9524–274,6429)	243.21 (200.4–281)	777	171.4 (98.2–231.4)	29	< 0.01*
S/F ratio at ICU admission		278.1 (156.6–332.1)	3942	150 (96–192)	69	< 0.01*
P/F ratio at ICU admission		154.5 (91–244.9)	3973	112.6 (79.9–150.5)	76	< 0.01*

*ICU* intensive care unit, *COPD* chronic obstructive pulmonary disease, *HIV* human immunodeficiency virus, *LDH* lactate dehydrogenase, *S/F* oxygen saturation to fraction inspired oxygen, *P/F* partial pressure of oxygen to fraction inspired oxygen

^*^P-value refers to statistically significant result

The inflammatory markers ferritin, D-dimer, NLR, procalcitonin, and LDH were higher in the group of patients who died and required IMV and increased progressively, were lower in outpatients, were increased in hospitalized patients, and reached the highest levels in patients admitted to the ICU and those who died in the emergency room, indicating significant differences in each of the groups. All these values were statistically significant with *p* < 0.05, where *p* = 0.05 is the limitation for statistical significance (Tables 1, 2, and 3).

The oxygenation indices evaluated were the S/F ratio and the P/F ratio. In the entire admission cohort, the median S/F was 339 (IQR, 310–423), and the median P/F ratio was 233 (IQR, 180–274). Among the patients who remained in the hospital for 48 h, the S/F ratio decreased by a median of 325 (IQR, 254–341). The median S/F ratio at ICU admission was 168 (IQR, 105–255), and the median P/F ratio was 118 (IQR, 81–171). In the ICU patients who died, the median P/F ratio was 105 (IQR, 76–146), and in those who survived, it was 137 (IQR, 91–198). The median P/F ratio at ICU admission in patients who underwent IMV was 112 (IQR, 79–150) and 154 (IQR, 91-244) in those who did not. It is clearly observed that as oxygenation indices fall, the possibility of dying, requiring IMV, and entering the ICU increases (Tables 1, 2, and 3).

### CART predictive model

Using the CART method, 2 predictive models were constructed, the first for IMV and the second for mortality.

A prediction model is presented in the CART methodology, which shows that a S/F ratio < 272 at 48 h of admission, high LDH levels at admission, and the presence of COPD are associated with an increase in the probability of requiring IMV. In the model, age ≥ 81 years decreased the possibility of the need for IMV, which is explained by the fact that many of these patients, due to personal decisions or clinical futility, were not taken to this therapy.

**Fig. 1:**
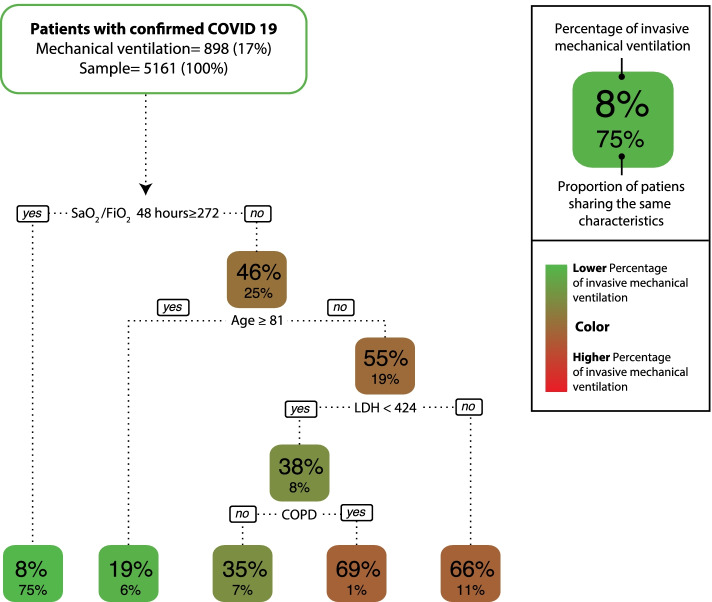
Need for invasive mechanical ventilation in patients with COVID-19, classification and regression tree (CART) risk groups: this method built a predictive model and five clinical profiles using the oxygen saturation to inspired oxygen fraction (SaO_2_/FiO_2_) ratio at 48 h after admission, age, lactate dehydrogenase (LDH), and chronic obstructive pulmonary disease (COPD) as predictor variables

This model shows how the S/F ratio at 48 h ≥ 272 gives a 92% probability of not requiring IMV during the hospital stay (Fig. 1).

**Fig. 2:**
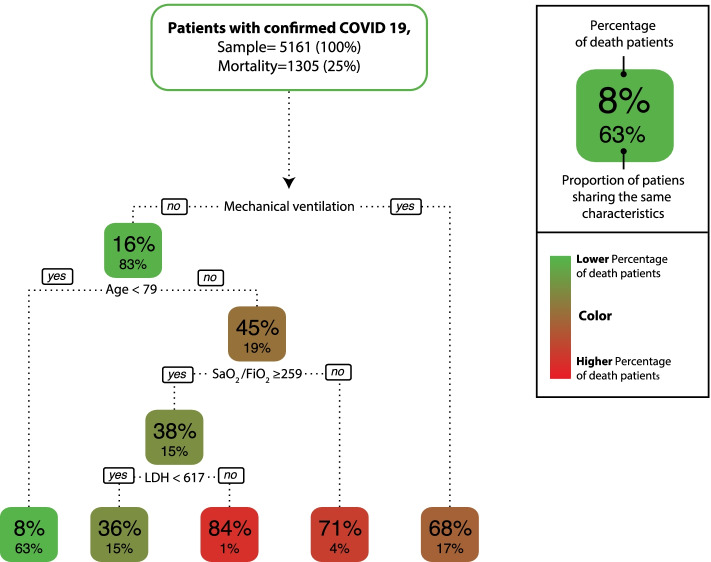
Mortality in patients with COVID-19, classification and regression tree (CART) risk groups: this method built a predictive model and five clinical profiles using the need for invasive mechanical ventilation (IMV), age, the oxygen saturation to inspired oxygen fraction (SaO_2_/FiO_2_) ratio on admission, and initial lactate dehydrogenase (LDH) as predictor variables

A prediction model is also presented using the same methodology, which shows that the main predictor of mortality in this cohort is the requirement of IMV. We see how age ≥ 79, S/F ratio < 259 at admission, and LDH level ≥ 617 U/L increase the probability of death (Fig. 2). In this model, patients with a S/F ratio greater than 259 at admission who also had an elevated LDH level (> 617 U/L) had a high probability of death, possibly indicating that the inflammatory response is a determining factor.

## Discussion

To our knowledge, this is the largest cohort of patients with COVID-19 living in a city located at an altitude of more than 2000 masl and one of the largest in a single centre.

The overall mean age in this cohort was 66 years (IQR, 53–77), which was one of the oldest reported [28–33], and 69 years (IQR, 59-76) in patients admitted to the ICU, which was the highest age reported in patients admitted to this service [33–37].

In all, 25% of the patients in this study died. If we excluded patients who were managed as outpatients and lost to follow-up, the mortality of patients requiring hospitalization was 30%, which is similar to other series reported in Italy (25%), the United States (US) (21%), Iran (24%), and Germany (27%) [30, 32, 38, 39] and higher than the series from China (5% to 16%) [28, 31, 40].

In this cohort, 1223 patients (24%) required ICU management, which was a larger proportion than the 7% found by Yang et al. in China [33] but similar to previous reports from the USA ranging from 22 to 32% [29, 37]. IMV was given to 898 patients (17%), which was a value higher than the Chinese studies, in which the use of IMV ranged from 1.4 to 8% [31, 40], and in an Italian study (8.7%) [30] but similar to studies from the USA in which 12.2%, 21%, and 22% of patients, respectively, required IMV [32, 37, 41]. This suggests that, in China, in-hospital management was given to patients with less severe disease.

Out of the 898 patients who received IMV, 613 of them died (68%), which is a figure lower than the studies from the onset of the pandemic in China (81%) [33] and the USA (88.1%) [41], which is similar to reports from the UK in March and April 2020 (66.3%) [42] and at the Brazilian National Registry of Intensive Care (66.9%) [43]. In later studies in high-income countries, the mortality reported in patients with IMV was lower, and among those, an Italian study showed mortality of 52% in ventilated patient s[36], and a multicentre prospective cohort of patients admitted to ICUs in France, Belgium, and Switzerland showed a 36% mortality rate [34].

Roedl et al. [44], in a multicentre study conducted in Germany, showed a mortality rate of 44% in patients with IMV. Lim et al. [45], in a review of 69 studies with more than 57,420 patients ventilated for severe COVID-19, found a mortality rate of 45% (95% *CI*, 39–52%); however, at the time of publication, many patients were still hospitalized. In this same study, the estimated mortality for patients aged 61 to 70 years was 77%.

Oxygenation indices have been broadly studied around the world as predictors of mortality and ICU admission in patients with COVID-19, leading to useful clinical practices to prioritize patients at hospital and ICU admission. Within those, a lower P/F ratio at ICU admission has been found to be a predictor of mortality among these patients, but few studies have been carried out regionally where this is stated.

In this study, the P/F ratio at ICU admission is one of the lowest when compared to other cohorts around the world, 118 vs. 129 in the USA; 160 in Italy; 154 in France, Belgium, and Switzerland; and 172 in China [29, 34, 35, 46]. This may be explained by the hypobaric hypoxaemia expected with altitude in Bogotá, Colombia. Although a study developed in critically ill patients in the USA at sea level [37] described a median P/F ratio of 103 at ICU admission, we analysed their parameters and found that the median FiO_2_ at ICU admission in these patients was 90%, compared to ours, in which the median FiO_2_ at ICU admission was 50%.

The P/F ratio on admission to the ICU in patients who underwent IMV was 112 vs. 154 in those who did not, thus indicating that the decision to start IMV was based not only on the oxygenation indices but also on the clinical presence of respiratory failure, and that a higher level of hypoxaemia was tolerated for the start of this therapy in relation to cities located at altitudes less than 1000 masl.

In patients admitted to the ICU, the P/F ratio was also significantly lower in patients who died (105) than in those who survived (137) compared to other studies where the P/F ratios in deceased patients and survivors were 134 and 163 [34], respectively.

There is insufficient clinical evidence to change the standard management of patients with COVID-19 based on oxygenation indices. The 2012 Berlin consensus [47] recommends an adjustment of the P/F ratio based on the BP in places over 1000 masl (BP/760); however, this recommendation does not have references on how it was derived [48]. In daily practice, based on experience and institutional management guidelines, patients with a P/F ratio greater than 250 without supplemental oxygen and without clinical dyspnoea may receive outpatient management. Patients with a P/F ratio of 200 to 250 are admitted to the hospital for surveillance, and supplemental oxygen is used for oxygen saturation greater than 88% and PaO2 greater than 60 mmHg. Patients with a P/F ratio less than 200 require surveillance in intermediate care with a high risk of requiring admission to the ICU. The decision to initiate IMV should not only be based on oxygenation indices but also on the presence of respiratory distress and tolerance and response to other methods of oxygen administration, such as high-flow cannula and noninvasive mechanical ventilation. Clinical trials are required to evaluate which oxygenation level once the patient is on mechanical ventilation is optimal for the initiation of neuromuscular relaxants and prone use. In the institutional guidelines of the Hospital Universitario Mayor, we recommend P/F levels of between 120 and 150 for these interventions at the discretion of the clinician.

Based on the current evidence, the oxygenation level at which it is best to start IMV is unknown. It must also be taken into account that during peaks of the pandemic, hospital occupancy was close to 100%, which interferes with the quality of care and may delay the start of IMV.

There is little information about the clinical characteristics of patients in high-altitude areas. Chen et al. [49], in a retrospective court of 67 patients who live in the Tibetan and Qiang Autonomous Prefecture of Ngawa, China, located at 2600 masl, found 4 severe cases (6%), 39 nonsevere cases (58.2%), and 24 (35.8%) asymptomatic cases, suggesting, as in the work of Seclén et al. [11], that altitude can protect against severe diseases caused by SARS-CoV-2. These results differ greatly from our findings, where only 17% of the patients were nonsevere cases with outpatient management, mortality was 25%, 24% of the patients were admitted to the ICU, and in 17% of the cases, IMV was used.

Abdelsalam et al. [50] analysed the clinical characteristics and laboratory findings of patients infected with COVID-19 in the city of Taif, Saudi Arabia, located at 1879 masl. Of 790 patients included in the analysis, 91.5% recovered without ICU admission, 6.8% recovered after ICU admission, and 1.7% died. In addition, a paired analysis was performed by age and comorbidities with 208 patients from the city of Jeddah, Saudi Arabia, located at sea level, finding a mortality of 14.4% and observing a lower mortality in people living at a high altitude. This reported mortality is much lower than that found in this study, which is explained by the age of the patients, since the total mean age was 41.4 years and 57.3 years among those who died, which is in great contrast with our population where the mean age of the entire population was 63.5 years and 74.3 years in the patients who died.

Several authors have documented alterations in C-reactive protein, D-dimer, and LDH that are associated with severity and mortality from COVID-19 [51]. Variations in the haemogram, coagulation tests, markers of myocardial injury, creatinine, and factors associated with systemic inflammation have been identified [52]. In the present study, the paraclinics that had a greater relationship with clinical outcomes in the bivariate analysis were LDH, D-dimer, and the neutrophil-lymphocyte ratio (NLR).

An uncontrolled inflammatory response is associated with worse clinical outcomes in SARS-CoV-2-infected patients [53]. Elevated serum concentrations of some inflammatory mediators have been described in patients with severe COVID-19, in particular, cytokines such as IL-1 and IL-6 [54]. However, in countries with limited resources, its clinical use is rare. More widely available tools, such as the NLR, can be used as markers of hyperinflammatory states [55, 56]. In COVID-19, it has been found that elevated NLR values are associated with a worse prognosis [57]. In addition, its ability to predict the severity and risk of death has been evaluated, as summarized in a meta-analysis that found an acceptable performance (mortality AUC 0.90 and severity AUC 0.85) with a cut-off of 6.5 [58].

Similar to the data described, our findings show a higher NLR among the deceased (NLR 11.78) than among the survivors (NLR 7.58). Likewise, the NLR was higher in patients admitted to the ICU (outpatient NLR 5.64, hospitalized NLR 7.82, and ICU NLR 11.58) and those who required IMV.

Moreover, the NLR among surviving patients was higher than those previously reported [59]. It remains to be confirmed whether high altitude can influence the baseline expression of inflammatory markers [60] or if it is a manifestation of disease severity at altitude. No previous articles have reported this ratio at high altitude.

LDH is the enzyme responsible for the passage from pyruvate to lactate, and its elevation has been associated with severity and death in infectious and neoplastic diseases [61, 62]. Different studies have established the performance of LDH as a prognostic biomarker in patients with SARS-CoV-2 infection [51, 63–65]. It has been documented that its elevation is associated with a risk up to six times higher for developing severe forms of COVID-19 (*OR* 6.7, 95% *CI*: 2.4–18.9) [63] and four times higher for mortality (*OR* 4.22, 95% *CI*: 2.49–7.14) [66]. In our study, the data were consistent with those previously reported. LDH was higher among the dead patients (471 U/L) than among the living patients (351 U/L). Likewise, its value was higher among ventilated patients (510 U/L) than among those who did not require this support (354 U/L).

Ballaz et al. [67] described the findings of the haemogram upon admission in Quito, Ecuador, at 2850 masl, finding expected inflammatory changes for patients with COVID-19, with a high NLR, and lymphopenia in the most severe cases. The study by Ballaz showed mean haemoglobin values similar to those in our study, 14.9 g/dL vs. 14.7 g/dL in nonsevere cases and 15.4 g/dL vs. 14.3 g/dL in severe cases. These findings suggest that haemoglobin values associated with exposure to high altitude at altitudes between 2500 and 3000 masl may not be relevant to the severity of COVID-19 presentation.

In the prediction model presented by the CART methodology, oxygenation indices, LDH level (as an inflammatory marker), and COPD (as a comorbidity) increase the probability of IMV, findings similar to other studies [37, 44].

In the prediction model for mortality, it is not surprising that the requirement for IMV is the main factor associated with death, since this indicates which patients are sicker; however, the model allows us to observe that being older than 79 years, hypoxaemia and elevated levels of LDH (as an inflammatory marker) increase the probability of death, even in nonventilated patients. These findings are similar to other studies [31, 33, 44, 46]. The advantage of this modelling is that it allows us to discover effect modifiers and complex interactions between variables.

## Conclusion

We conclude that, although the oxygenation indices are lower in the city of Bogotá due to hypobaric hypoxaemia, the need for hospitalization, admission to the ICU, and IMV are similar to other regions less than 1000 masl, except China, where most data indicate that patients with less severe disease were hospitalized.

The mortality of patients with IMV in this study is high but similar to that reported in high-income countries at the beginning of the pandemic and in other Latin American countries; however, it has remained high throughout the entire pandemic, unlike high-income countries where it has decreased, suggesting hypobaric hypoxaemia may not be the main factor in mortality from COVID-19 leading to IMV, and the availability of resources is an important factor in mortality.

In this study, it was also found that the levels of oxygenation indices at altitude, which is management decisions such as the start of IMV and therefore associated therapies (e.g. use of neuromuscular relaxants and prone), are lower than at sea level; however, clinical trials should be performed to determine the P/F ratio cut-off point of greatest benefit for these therapies.

### Limitations

This study has many limitations. It is a descriptive study of a single-centre high complexity hospital in the city of Bogotá, which may not reflect the behaviour of the pandemic throughout the country. It does not describe the pharmacological therapies used in the patients; however, it is noted that, in Colombia, in the drugs with some evidence of benefit in clinical use in patients with COVID-19 for general use, only dexamethasone in patients admitted to the hospital with supplemental oxygen was available (no other drug therapies were available (e.g. remdesivir and tocilizumab). Also, the vaccination effort began at the end of March 2021, so this study does not yet reflect the impact of this measure. The use of other ventilation therapies, such as noninvasive and high-flow cannulas, is not described, since these were used outside of the ICU and in expansion areas, and the quantification of their time of use given that the records in the clinical history were not performed exactly.

## Data Availability

All data used and analysed during this study are available from the corresponding author on reasonable request.

## Abbreviations

COVID-19: Coronavirus disease 2019
SARS-CoV-2: Severe acute respiratory syndrome coronavirus 2
masl: Metres above sea level
IMV: Invasive mechanical ventilation
CART: Classification and regression trees
IQR: Interquartile range
ICU: Intensive care unit
PaO_2_: Pressure arterial oxygen
FiO_2_: Fraction inspired oxygen
P/F: Ratio of partial pressure arterial oxygen to fraction inspired oxygen
SaO_2_: Oxygen saturation
S/F: Ratio of oxygen saturation to fraction inspired oxygen
LDH: Lactate dehydrogenase
BP: Barometric pressure
PiO_2_: Pressure of inspired oxygen
ACE: Angiotensin-converting enzyme
RT-PCR: Real-time reverse transcription-polymerase chain reaction
BMI: Body mass index
NLR: Neutrophil-lymphocyte ratio
ED: Emergency department
RPART: Recursive partition and regression trees
COPD: Chronic obstructive pulmonary disease
AUC: Area under the curve
